# The Effect of Encapsulation Geometry on the Performance of Stretchable Interconnects

**DOI:** 10.3390/mi9120645

**Published:** 2018-12-05

**Authors:** Mahmoud Mosallaei, Jarno Jokinen, Mikko Kanerva, Matti Mäntysalo

**Affiliations:** 1 Laboratory of Electronics and Communications Engineering, Faculty of Computing and Electrical Engineering, Tampere University of Technology, 33720 Tampere, Finland; matti.mantysalo@tut.fi; 2 Laboratory of Materials Science, Faculty of Engineering Sciences, Tampere University of Technology, 33720 Tampere, Finland; jarno.jokinen@tut.fi (J.J.); mikko.kanerva@tut.fi (M.K.)

**Keywords:** encapsulation, finite element analysis, printed electronics, screen printing, stretchable interconnects

## Abstract

The stretchability of electronic devices is typically obtained by tailoring the stretchable interconnects that link the functional units together. The durability of the interconnects against environmental conditions, such as deformation and chemicals, is therefore important to take into account. Different approaches, including encapsulation, are commonly used to improve the endurance of stretchable interconnects. In this paper, the geometry of encapsulation layer is initially investigated using finite element analysis. Then, the stretchable interconnects with a narrow-to-wide layout are screen-printed using silver flake ink as a conductor on a thermoplastic polyurethane (TPU) substrate. Printed ultraviolet (UV)-curable screen-printed dielectric ink and heat-laminated TPU film are used for the encapsulation of the samples. The electromechanical tests reveal a noticeable improvement in performance of encapsulated samples compared to non-protected counterparts in the case of TPU encapsulation. The improvement is even greater with partial coverage of the encapsulation layer. A device with a modified encapsulation layer can survive for 10,000 repetitive cycles at 20% strain, while maintaining the electrical and mechanical performance.

## 1. Introduction

Stretchable electronics have attracted a large amount of attention in the last few years, since they can bring freedom of design in a variety of applications [1]. A typical stretchable device has the ability to deform into arbitrary shapes without interrupting its functionalities, either electrical or mechanical [2]. This outstanding feature has been shown in a range of applications, such as soft robotics [3], wearable electronics [1], health monitoring systems [4], stretchable smart displays [5], and soft sensors [6]. Unlike deformable materials, the inherent mechanical limitation of conventional rigid components is a major issue, especially for skin and wearable devices [7]. The unique mechanical properties of stretchable devices would allow using them on skin or textile, where they can conform to the uneven surface of a human body without using rigid, large, and heavy devices that would make them noticeable and uncomfortable for users [4,7].

One common method for making a stretchable device is to use the island-bridge technique, because of its ability for large and reversible stretchability [8]. In this approach, functional units are miniaturized while they are still rigid. These units are connected together using stretchable interconnects and wiring [7,8,9]. The stretchability of interconnects plays an important role in the deformability of the whole system, since islands can stay unchanged only for a very small strain [3,10,11]. Based on the type and functionality of the device, the conductivity should be maintained either during stretching, or when they return to the initial state. A number of parameters can affect the robustness of interconnects; these include the material, adhesion between the substrate and the conductive coating, and the geometry of the conductive material [12].

The proper materials for making deformable electronics should satisfy a number of requirements [13]. The materials for the substrates, electrical conductors, adhesive layers between components and the substrate, and the protective layer, all have different physical, mechanical, and chemical properties compared to each other [3]. Considering these differences, combining these materials into a single device is challenging, especially from the mechanical point of view [13].

Plastics and elastomers, such as polyurethanes (PUs) and polydimethylsiloxane (PDMS), are commonly used as substrate, due to their good properties [13,14]. Generally, PDMS is more commonly used than PUs for stretchable electronics. Both PDMS and PUs are very stretchable, light-weight, present high elongations at break [15], and have a low Young’s modulus, which are advantages—especially in wearable devices—since they improve the comfort and unobtrusiveness of the device for the user [14,15]. Furthermore, elastomers are inert, which makes them good candidates for skin-mounted applications. In addition, they are relatively cheap, which is beneficial in mass production [16].

There are two types of materials for making conductive components [17]. The first group consists of intrinsically soft materials, such as conductive polymers (e.g., polyaniline) [18], liquid metals (e.g., Eutectic Gallium-Indium(EGaIn)) [2], and conductive composite materials (e.g., multi-walled carbon nanotubes (MWCNTs)/PU composite) [19,20]. Although these materials show some level of mechanical flexibility, their conductivity level is limited [9]. The other group includes rigid solid metals and inorganic compounds. Metals are conventionally used in electronics due to the low electrical resistivity and high thermal conductivity. The major issue is that metals in bulk form are not especially flexible and stretchable, due to their high Young’s modulus and very low stretchability (<1%) [20,21]. However, specific geometries, such as a U-shape, a horseshoe, a zigzag, and a multi-track pattern, can improve the stretchability of thin conductive track by utilizing the bending of the system into the third dimension [22,23]. By tailoring the geometry, load can have less effect on conductive tracks and, thus, the reliability of the device is improved [24,25].

In addition to materials and engineered design, encapsulation is needed to protect the device. The goal is to protect functional units and interconnects from external stimulus. This is more important when the device functions in harsh environments, like a humid atmosphere, ionic contamination, radiation exposure, a vibrating situation, or continuous mechanical stresses (e.g., loading and unloading) during the operation life [26].

In this paper, the effect of encapsulation geometry on the electrical and electromechanical performance of stretchable interconnects is investigated. For this aim, finite element (FE) analysis was initially performed to find the optimum dimensions and geometry for the encapsulation layer. Stretchable interconnects were manufactured by the screen-printing technique. The effect of using UV-curable screen-printed dielectric ink and heat-laminated TPU film as an encapsulation layer were investigated. The electromechanical behaviors of samples were compared. To mimic the real-life performance of the interconnects, repetitive cyclic strain tests were used.

## 2. Materials and Methods

### 2.1. Materials

We used a 50 µm-thick transparent TPU sheet from Epurex Platilon (4201 AU, Epurex Films GmbH & Co. KG, Lower Saxony, Germany) in this study. This sheet was employed for both substrate and encapsulation film. This TPU exhibits high electrical resistance and high strain to failure (over 300%). However, the non-linear stress–strain behavior of the TPU, especially at higher stretching values, is challenging from the analysis point of view. For making the interconnects, a highly conductive, commercially available silver paste (CI-1036, Engineered Conductive Materials, LLC, Delaware, OH, USA) from engineered conductive materials (ECM) was used, which is a composite of silver flakes in polymer matrix. Screen-printed silver flakes form relatively thick conductive layer (range 7–15 µm, depending on the screen) resulting in a low sheet resistance. The total solid content of this ink is 66%, and the viscosity and density are 10,000 mPa∙s at 25 °C and 2.08 kg/L, accordingly. We also used a UV-curable dielectric ink (DI-7540, Engineered Conductive Materials, LLC) from ECM as an encapsulation layer. This ink has the same viscosity as the conductive paste, and the total solid content is 100%. The density of this dielectric ink is 1.27 kg/L, and the electrical resistance is over 1 GΩ. This ink was deposited by the screen-printing process.

### 2.2. Fabrication

**Figure 1:**
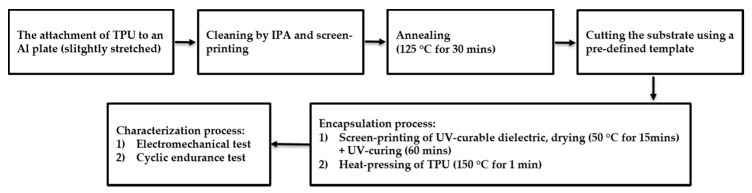
A summary of the fabrication and characterization of stretchable interconnects.

A screen-printing method was used to fabricate the conductive track and dielectric overlayer. The screen used in this work was fabricated by Finnseri Oy (Hyvinkää, Finland). The screen contains polymer mesh with a 79 threads/cm mesh count, 69 µm mesh opening, and 55 µm thread diameter. Before the printing process, the TPU substrates are slightly (approximately 3%–5%) stretched, and attached to aluminum plates. The aluminum plate supports the sheet to prevent folding during the printing process. Semi-automatic printing equipment (TIC, SCF-300, Siebdruck-Service Eickmeyer GmbH, Bünde, Germany) was used for the printing process. For the fabrication of conductive tracks, two consecutive printing cycles were performed. The conductors were heat-annealed in oven for 30 min at 125 °C. The printing quality was inspected by using an optical microscopy. Two different encapsulation methods were investigated: (1) UV-curable dielectric ink and (2) heat-laminated TPU. A screen-printable dielectric, as the encapsulation layer, was printed on interconnects after properly aligning the substrate under the screen. Samples were initially dried in an oven for 15 min at 50 °C. Then, the UV radiation provided by Aktina S UV-exposure unit (Walter Lemmen GmbH, Kreuzwertheim, Germany), which cured them for one hour. The exposure dosage of the unit is approximately 3.3 mJ/cm^2^, and the type of lamp is Philips TL-DK 30W/10 Actinic BL (Philips, Amsterdam, Netherlands). However, in this unit, the UV intensity was constant, and the UV-exposure time for the dielectric ink was found experimentally. For the heat encapsulation, TPU sheets were cut in different layouts and a normal heat press machine (Combo Heat Press, Dongguan STC Machinery Co. Ltd, Dongguan, China) was used to perform heat lamination. The heat lamination process was 150 °C for one minute. In both encapsulation methods, interconnect pads were not covered to enable electrical measurement. The thickness of the TPU substrate, the conductive path, and the dielectric ink are 50, 10, and 18 µm, respectively. Samples were then cut into the desired rectangular shape size of 142 mm × 38 mm, for the characterization stage. Figure 1 shows the flowchart of the fabrication steps of stretchable interconnects, as well as the characterization methods.

**Figure 2:**
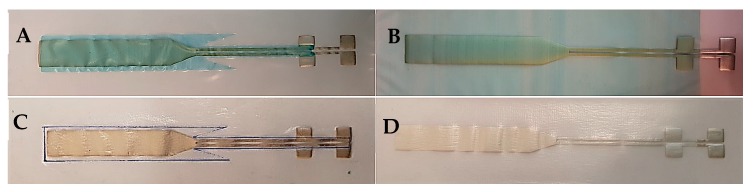
The screen-printed stretchable interconnects with different encapsulation materials. (**A**) Partially dielectric ink-coated, (**B**) entirely dielectric ink-coated, (**C**) partially thermoplastic polyurethane (TPU) heat-laminated, and (**D**) entirely TPU heat-laminated interconnect.

Figure 2 represents the fabricated stretchable interconnects encapsulated partially and entirely by either dielectric ink (2A, 2B) or TPU film (2C, 2D).

### 2.3. FE Analysis

**Figure 3:**
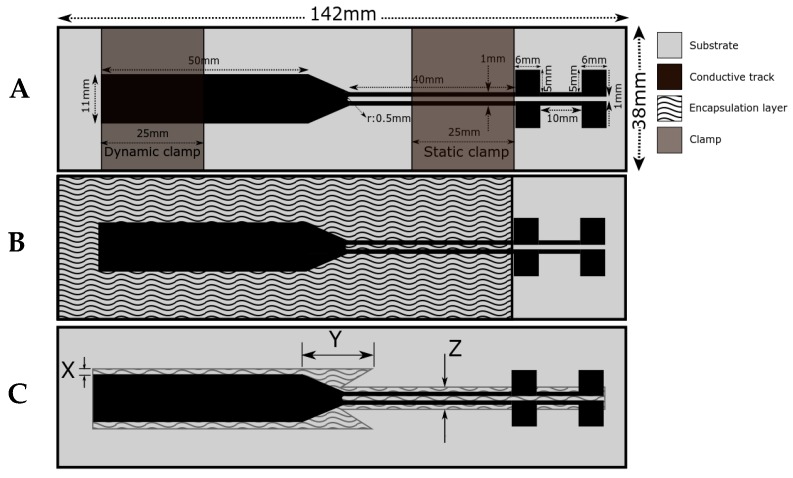
A schematic of stretchable interconnects. The dimensions of the substrate, conductive track and clamps are the same for all cases. (**A**) a non-encapsulated sample (Set 1). (**B**) An entirely encapsulated sample (Set 3 and Set 5) (except for the pads used for electrical characterization) corresponding to Figure 2B,D. (**C**) A partially encapsulated sample (Set 2 and Set 4) (X, Y, and Z are offsets) corresponding to Figure 2A,C.

The target of the FE analysis was to optimize the geometry of the encapsulation layer for improving the performance during the stretching. The FE model was created using the commercial FE software Abaqus 2017 (Simulia, Dassault Systemes, Johnston, RI, USA). The FE model was created using solid elements. The substrate, coating, and TPU encapsulation layer were modeled as separate parts. All parts were attached to each other using a tie constraint. The geometry of the conductive path and substrate were kept constant in all of our analyses, while the geometry of the dielectric was modified. Figure 3A shows the schematic of stretchable interconnect and the location of both static and dynamic clamps. Figure 3B represents the schematic of an entirely encapsulated sample (by either TPU or dielectric ink) while, in Figure 3C, samples are partially encapsulated. The geometry modification was defined with three parameters (X, Y, and Z), as depicted in Figure 3C.

The FE analysis was performed for a system, including the TPU dielectric as the encapsulation layer. The dielectric and substrate were defined as the same material. The substrate and conductive material properties were fitted based on the reverse engineering according to our previous work [12]. The TPU substrate was modeled using a hyperelastic material model (Ogden *n* = 1) provided by stress–strain data obtained from the uniaxial tensile test. The conductive component Young’s modulus and Poisson’s ratio were 550 MPa and 0.035, respectively. The applied material model for the conductive component was elastic-plastic. Two points defined plasticity: the first point was at a yield strength of 15 MPa with 0 plastic strain, and for the second point, the stress level was 80 MPa with 1% plastic strain [12].

**Figure 4:**
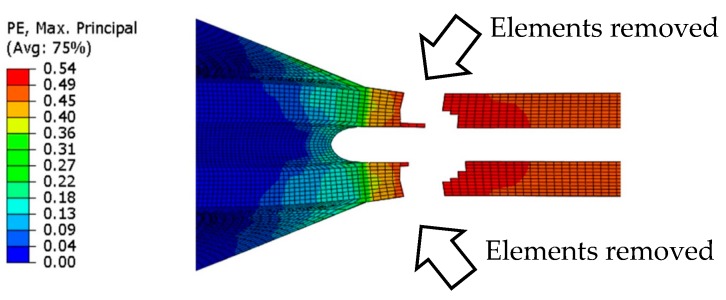
The failure zone on the conductive track after the critical plastic strain (PE) limit.

In this work, the failure of the conductive film was introduced. The finite element of the conductive film was assumed to have failed when the critical plastic strain was exceeded. The critical plastic strain was defined based on the conduction failure of the reference experiment (shown in Table 1). The conductivity was assumed to have failed when the elements did not form a continuous path (as shown in Figure 4).

Figure 4 presents the area close to the connection where elements exceed the critical plastic strain. This area was removed for the visualization purposes. The same experimental conductive failure in the finite element model was reached when the critical plastic strain was 52.5%; this was used in our further analysis.

The boundary conditions of the specimen were similar to those in our previous work [12]. The location of clamps was as shown in Figure 3A. Boundary conditions of the specimen were defined in the location of clamps. All three displacements (including out of plane displacement) were restricted at the one side of the specimen (static clamp). At another side of the specimen, the longitudinal displacement was not restricted while two other displacements, normal to the longitudinal displacement (width and thickness direction), were restricted (dynamic clamp). The enforced displacement was added at a magnitude of 50 mm. However, the analyses were aborted when failure occurred, because the definition of the failure of the coating was our point of interest. Analyses were performed in a piecewise manner, and failure was dependent on incrementation. The maximum increment was limited to 1% in the analysis.

**Figure 5:**
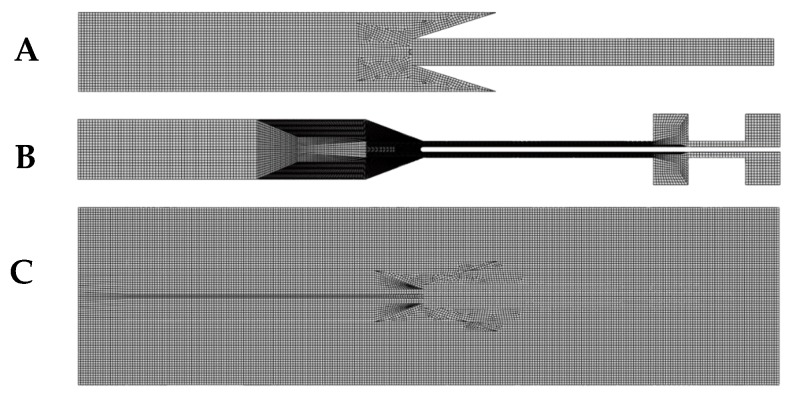
The meshing system for (**A**) the encapsulation layer, (**B**) the conductive path, and (**C**) the substrate.

FE analyses are mesh-sensitive and the mesh was kept as constant as possible. The finite element mesh of the conductive material was not modified during the analysis. The substrate mesh was not constant in all analysis. The changes were caused by the partition defining the tie constraint between the substrate and the encapsulation layer. The partition was modified according the geometry changes in the encapsulation layer. The substrate and the encapsulation layer were meshed using C3D8H elements, and the typical element dimension was 0.5 mm. The conductive path was meshed using a C3D8R element, with the typical dimension of 0.5 mm. The mesh was finer at the junction than in the rest of the coating. The typical element mesh for three parts is shown in Figure 5.

## 3. Characterization Methods

**Figure 6:**
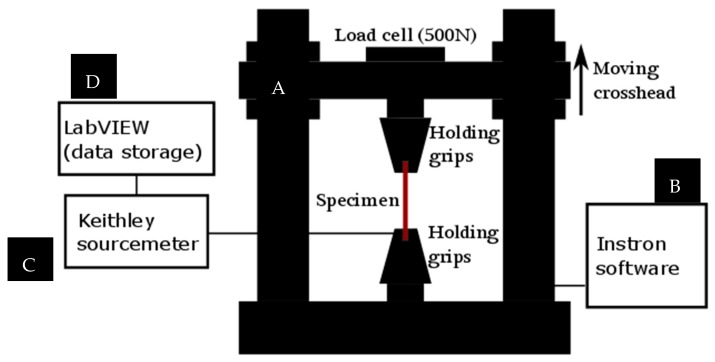
The schematic of the setup for electromechanical assessment. (**A**) The Instron 4411 Universal Testing Machine, (**B**) Instron software on a PC, (**C**) Keithley 2425 SourceMeter, and (**D**) LabVIEW software (version 2014, National Instruments, Austin, TX, USA) on a laptop.

We used an Instron 4411 Universal Testing Machine (Instron, Norwood, MA, USA) for the mechanical characterization of the specimen. Different specimens with/without an encapsulation layer in different shapes were analyzed accordingly. To place the specimens in the machine, customized clamps were used. These clamps were partially coated by a thin layer of rubber in a way that the conductive path is not contacted by the metallic part of clamps when they are closed, thus preventing early damage of the conductive path. The rubber film on the grip faces increases the coefficient of friction, and prevents failure in the grip section. Hence, possible slippage and failure in the clamp section were excluded. Figure 6 shows the schematic of the electromechanical assessment equipment.

Pull-up speed, sample rate, and grip distance were adjusted to 6 mm/min, 5 Hz, and 50 mm, respectively. A load sensor of 500 N was used, since it can precisely measure the small forces from the stresses placed upon the cross-section of delicate substrates, like TPU. Pneumatic pressure was used to close the clamps. After a measurement, the system software provides mechanical data, such as force versus displacement.

The initial electrical resistivity of the interconnects was measured by a Keithley 2425 resistance measurement device (Keithley Instruments, Cleveland, OH, USA). To assess the resistance change of the interconnects for either a single pull-up test or during a cyclic test, the interconnects were inserted into an Instron machine, and their resistances were measured by a Keithley SourceMeter (Keithley Instruments, Cleveland, OH, USA), during the deformation, simultaneously. The interconnect pads were attached to a rigid piece of printed circuit board (PCB) with a layer of anisotropic conductive film (ACF), and this configuration is connected to the SourceMeter by wires. We used a two-probe measurement system in this work. To minimize the noise in contact resistance, interconnection pads were under the static clamp. Real-time resistance measurement was performed during the stretching and cyclic tests. After performing the tests, corresponding values for displacement and resistance (or the number of cycles and resistance) were matched together. To simulate the long-life performance of the interconnects, cyclic tests were performed for 10,000 cycles for different stretching values (10%, 20%). To speed up the process, a higher cyclic speed (200 mm/min) was chosen.

## 4. Results

### 4.1. FE Analysis

In total, 23 geometries of the TPU encapsulation layer were studied. The different cases are shown in Table 1. The references geometry for defining the critical plastic strain parameters were 25, 2, and 5 mm, for Y, X, and Z, respectively. The range of failure strain was from 50% to almost 60%.

Typically, the failure initiation of the conductive material did not immediately cause the final failure of the conductivity in FE analysis. The failure of some geometries was more sudden than others. The failure initiation typically existed at the sides of the conductive lines. Geometry parameters have an interaction and cannot be studied independently. The reference case (in Table 1) was shown to provide the best failure strain. For that reason, it was chosen to be used in experiments presenting the partially TPU-encapsulated specimen. FE analysis was also performed for the entirely TPU-encapsulated specimen. The entirely TPU-encapsulated specimen provided 38.3% failure strain, which was lower than the partially TPU-encapsulated specimen, but it was close to minimum boundary of the experimental deviation. The entirely encapsulated FE model does not influence on strain distributions in the same way as the partially TPU-encapsulated FE model. The difference between the partially and the entirely TPU-encapsulated specimens can be explained by strain distributions. The partially TPU-encapsulated strain distribution was spread more widely than in the entirely TPU-encapsulated specimen. The failure was located in the transition area where the narrower line transforms to the wider part in both entirely and partially TPU-encapsulated specimens.

### 4.2. The Peel Test

**Figure 7:**
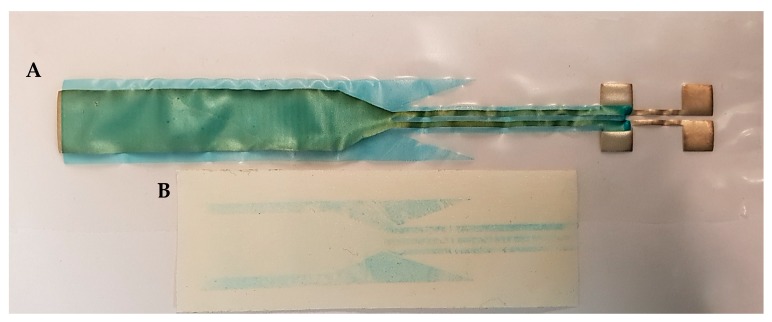
Peel test result. (**A**) The same sample after performing the peel test; (**B**) Tape used for the test. The blue trace indicates the poor adhesion between the substrate and the dielectric ink.

A peel test was performed to evaluate the adhesion strength of the components before performing the tensile and the endurance cyclic test. For this aim, the adhesion strength between the dielectric ink and the conductive path, as well as the dielectric ink and the TPU substrate, were evaluated. In this test, the tape in standard ASTM-D3359 (a standard test methods for measuring adhesion by tape test) was used; however, it was not possible to make a crosscut due to the soft nature of the substrate. Figure 7 shows the result of the peel test for a sample encapsulated by the dielectric ink.

After the tape was removed, we found out that, although there was a robust adhesion made between the dielectric and the conductive path, the adhesion between the dielectric ink and the TPU substrate was quite weak, which can negatively influence on the protective effect of the dielectric on the conductive path. The same experiment for evaluation of adhesion strength between the conductive path and the TPU substrate revealed a robust adhesion between these two components.

### 4.3. The Tensile Test

The stretchability of interconnects was defined by stretching samples until conductivity was lost. Ten specimens from each sample set of interconnects were unidirectionally stretched and compared to non-encapsulated samples, in order to see the effect of encapsulation and encapsulation’s geometry. Samples with partial encapsulation were prepared based on the results obtained from the FE analysis. All sample sets are described in Table 2.

**Figure 8:**
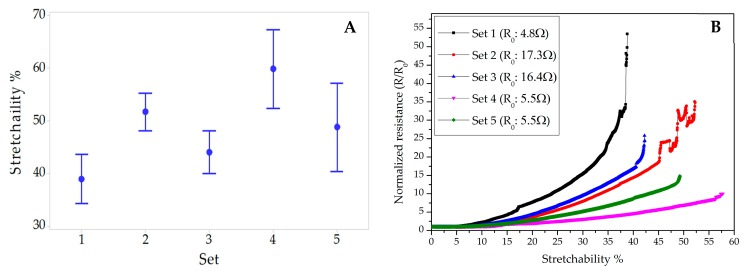
The stretchability feedback of interconnects. (**A**) An interval plot of the stretchability of different sets of samples: (1) non-encapsulated, (2) partially ink-encapsulated, (3) entirely ink-encapsulated, (4) partially TPU-encapsulated, and (5) entirely TPU-encapsulated; the mean values are 38.9%, 51.7%, 44.01%, 59.84%, and 48.81%, respectively. (**B**) Comparison of normalized resistances of a typical performance of each samples. Initial resistances (R_0_) are 4.8, 17.3, 16.4, 5.8, and 5.5 Ω for sets 1–5, respectively.

Figure 8A shows the interval plot of the stretchability of samples that were non-encapsulated (Set 1), partially (Set 2), and entirely (Set 3) dielectric ink-encapsulated, and partially (Set 4) and entirely (Set 5) TPU-encapsulated. Figure 8B shows a typical performance of each sample sets.

As it is illustrated in Figure 8B, the initial resistances of the samples encapsulated by UV-curable dielectric ink increased about three times after the encapsulation process. To analyze this change, first a non-encapsulated sample was UV-cured for one hour (similar to the curing process of the dielectric ink). After the UV-curing process, it was found out that the UV exposure did not have any influence on the initial resistance of the sample. Then a sample encapsulated by dielectric ink was observed by an optical microscope and it was realized that small microcracks emerged on the surface of the conductive path after the curing process. These microcracks caused an increase in the initial resistances of all samples encapsulated by the dielectric ink.

The FE analysis of the entirely TPU-encapsulated sample provides lower failure strain than experiments, but it is close to the minimum boundary of the experimental deviation. The entirely encapsulated FE model did not influence on strain distributions in the same way than the partially TPU-encapsulated FE model. The maximum value for the stress and strain is concentrated in the transition area where the narrower line transforms to the wider part (Figure 4, area where elements are removed). The FE model was not able to provide a similar result when adding data from entirely TPU encapsulation. This could be partly explained by the inability of FE to model protection provided by the encapsulation. In our previous study, the stress concentration points on the transition area of the conductive path was shifted successfully by adding so-called sacrificial edges on the sides of the transition area. Consequently, the stress concentration zone shifted to these edges, where the conductivity was not affected and, therefore, the electromechanical performance was improved [12]. In this study, the same way of improvement was investigated by making sacrificial edges out of the encapsulation layer. Generally, encapsulation of the stretchable interconnects protects the sample from mechanical stresses by restricting the initiation and propagation of microcracks on the conductive path (delayed crack propagation). This can be seen in the improvement of the stretchability of all the entirely encapsulated samples compared to their non-encapsulated samples. The stretchability can be further enhanced by partial encapsulation to locally increase the stiffness. Subsequently, when both entirely and partially encapsulated samples are stretched up to the same stretching value, less strain went to the encapsulated areas where we locally increased the stiffness because the stress was first released by the non-encapsulated areas. To find a partial geometry for encapsulation layer, we included the sacrificial edges obtained from FE analysis in its layout (shown by parameter Y in Figure 3C), since shifting the stress concentration area from the transition area can be done in a more efficient way compared to the other geometries shown in Table 1.

**Figure 9:**
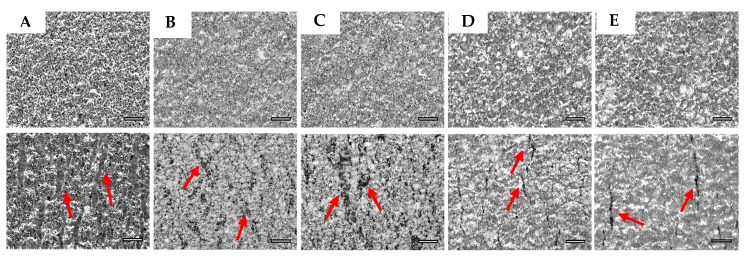
Optical microscope images of the transition area from all sets of samples (sets 1–5 are marked by **A**–**E**) when they are not stretched (upper images) and when they are stretched up to 60% (lower images). The scale bar in all images is 100 μm.

Optical microscope images were taken from the transition area (Figure 4 where the elements are removed) for different samples to understand the effect of both the encapsulation and the encapsulation geometry on tensile performance. All samples were unidirectionally stretched up to 60% to compare the crack magnitude and the crack density of this area. As Figure 9 shows, there is a continuous conductive path for all samples when they were not stretched (upper images). After samples were stretched up to 60%, the formation of the microcracks and their magnitude were different for each set of samples. Arrows show some points where microcracks emerged. The non-encapsulated sample presents the higher density of microcracks. For the samples encapsulated by the dielectric ink, higher nucleation areas for microcracks can be seen. On the other hand, the population of the microcracks and their magnitude are less for samples encapsulated by TPU; however, microcracks are linked together and make bigger cracks for samples entirely encapsulated by TPU in comparison with when TPU is partially encapsulated. The microscope images correspond well with the result of the tensile test for different sets of samples.

### 4.4. The Cyclic Test

**Figure 10:**
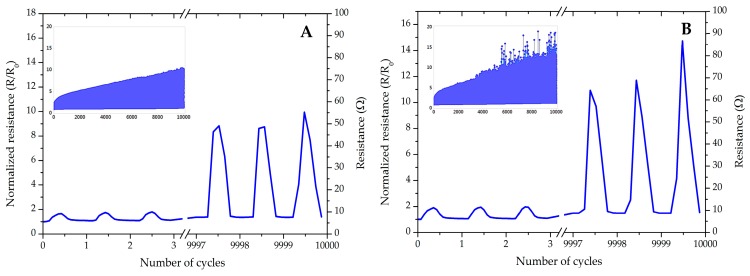
The normalized/normalized resistances during 10,000 cycles at 10% stretching. (**A**) Partially TPU-covered, R_0:_ 5.4 Ω. (**B**) Entirely TPU-covered, R_0_: 6.1 Ω. The insets are the overall trend during all the cycles.

The endurance of interconnects during the cyclic test was investigated. We selected the samples encapsulated by the TPU layer due to their better reliabilities. Figure 10A,B show the three first and last cycles of the normalized/absolute resistances of the interconnects encapsulated, either partially or entirely by TPU, during 10,000 repetitive stretching–releasing cycles at 10% stretching. The inset images present the overall trend in changing of the resistances during all cycles.

**Figure 11:**
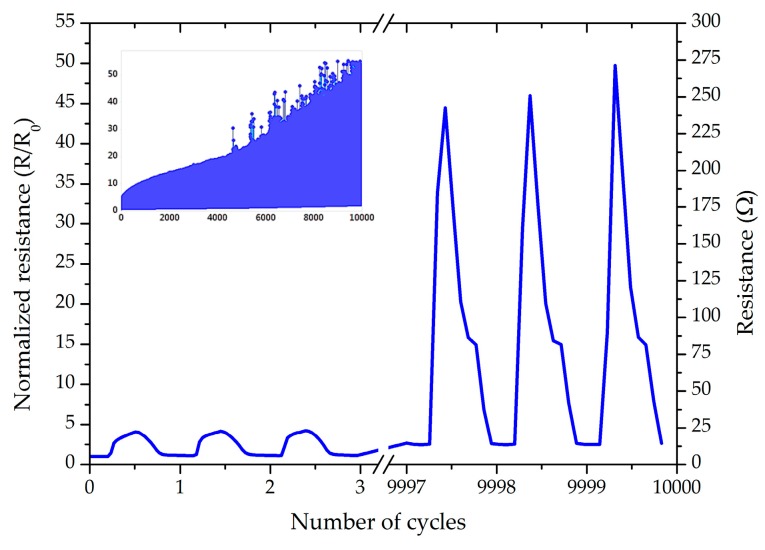
The normalized/absolute resistances of the partially TPU-covered samples at 20% stretching, R_0_: 5.5 Ω. The inset presents the overall trend during all the cycles.

As can be seen, both samples could resist 10,000 cycles with 10% stretching; however, the growth of resistance is less for partially coated samples. Figure 11 presents the cyclic test for a partially TPU-encapsulated for 10,000 cycles and strain level of 20%.

Unlike the entirely TPU-encapsulated sample, the partially TPU-encapsulated sample could resist 10,000 cycles at 20% strain without losing conductivity, which proves our hypothesis about the superiority of encapsulation geometry modification discussed in Section 4.2, even in the long-term endurance of the interconnects. The cyclic endurance result for the samples encapsulated by the dielectric ink were, however, weak. The first reason is the poor adhesion strength between the TPU substrate and the dielectric ink, which did not provide a protective support against the mechanical deformation for the inner conductive path. We also analyzed the effect of UV exposure on the cyclic performance of the samples. For this aim, a non-encapsulated sample was exposed to UV radiation, similar to the curing process of the dielectric ink. We performed the cyclic test at 20% strain and the sample failed electrically after 819 cycles, which is 503 cycles less than the average of non-encapsulated sample at 20%. This phenomenon is a result of mechanical degradation of the substrate by the effect of UV radiation, which leads to a reduction in physical and mechanical properties of the TPU, and weakening of the bonding strength between the TPU substrate and the conductive path. TPU also becomes yellowish because of the oxidation reaction in the UV chamber. A combination of the weak adhesion strength of the dielectric ink and the TPU substrate, and the destructive effect of the UV radiation on the TPU substrate, led to the weak performance of the UV-encapsulated samples during the cyclic endurance test. Table 3 is a summary for the number of cycles before the electrical failure for each sets of samples.

## 5. Summary and Outlook

The functionality of deformable electronic devices during service life is an important aspect that is continuously improving. Stress distribution on substrate is important in delamination, crack initiation, and crack propagation on the conductive path when the device is subjected to stress, and these can lead to the early failure of the devices. One common way to enhance the reliability and lifespan of these devices is protecting them by encapsulation layers. Therefore, the nature and geometry of the encapsulation layer play an important role against deformation. 

In this paper, the effect of encapsulation geometry on the deformation, failure, and reliability mechanisms of stretchable interconnects was investigated. For the electrical conductivity to transit from a narrow format to a wide format, the layout from our previous work was chosen [12]. Although this layout showed 13% less stretchability than the sample with the modified design, less conductive materials were used in the fabrication. Less required material would lead to a considerable economical saving in large productions. This benefit led us to use the same layout and improve the functionality by the application of an encapsulation layer. For this aim, a set of geometries for the TPU encapsulation layer were initially investigated by FE analysis, and samples with better geometry, including sacrificial zones, were fabricated alongside entirely coated and non-encapsulated samples, for comparison. Stretchable interconnects were screen-printed using deformable materials. Two different dielectric materials were applied by screen-printing and heat lamination techniques. The samples with encapsulation layers showed considerable improvement in a single tensile test when compared to the non-encapsulated samples. In addition, by removing the excess dielectric layer around the conductive path and tailoring the dielectric geometry, the stretchable interconnects performed better than entirely covered samples, because of the stress released by the non-coated area and sacrificial zones, that was earlier than on the conductive path. This retards the initiation, propagation, and density of microcracks on the conductive path, and improves functionality. The effect of encapsulation was also noticeable in the cyclic behavior of samples. Interconnects with an engineered design could stand 10,000 cycles and 20% stretching, while maintaining conductivity and mechanical integrity.

## Figures and Tables

**Table 1:** Finite element (FE) analysis results for different encapsulation layer geometric parameters (see Figure 2).

Y (mm)	X (mm)	Z (mm)	Failure Strain (%)
15	1	5	55.3
20	1	5	58.1
25	1	5	56.2
30	1	5	54.1
15	1	7	50.5
20	1	7	53.1
25	1	7	52.1
30	1	7	50.9
15	2	5	51.8
20	2	5	59.2
25	2	5	59.8 ^1^
30	2	5	56.6
15	2	7	51.0
20	2	7	54.6
25	2	7	54.0
30	2	7	52.3
15	3	5	54.0
20	3	5	51.2
30	3	5	59.2
15	3	7	53.0
20	3	7	56.3
25	3	7	56.1
30	3	7	54.4

^1^ Reference case.

**Table 2:** The description of all sample sets corresponded to the Figure 3.

Sample Set	Description	Geometry
Set 1	Non-encapsulated sample	Figure 3A
Set 2	DI-7540 partially encapsulated	Figure 3C (X: 2 mm; Y: 25 mm; Z: 5 mm)
Set 3	DI-7540 entirely encapsulated	Figure 3B
Set 4	TPU partially encapsulated	Figure 3C (X: 2 mm; Y: 25 mm; Z: 5 mm)
Set 5	TPU entirely encapsulated	Figure 3B

**Table 3:** Number of cycles before the failure (sets 1–5).

Stretch %	Set 1	Set 2	Set 3	Set 4	Set 5
10	1915	322	286	10K	10K
20	1322	181	109	10K	6983

## References

[1] Yokus M.A., Foote R., Jur J.S. (2016). Printed stretchable interconnects for smart garments: design, fabrication, and characterization. IEEE Sens. J..

[2] Dickey M.D. (2017). Stretchable and soft electronics using liquid metals. Adv. Mater..

[3] Yu X., Mahajan B., Shou W., Pan H. (2016). Materials, mechanics, and patterning techniques for elastomer-based stretchable conductors. Micromachines.

[4] Do T.N., Visell Y. (2017). Stretchable, twisted conductive microtubules for wearable computing, robotics, electronics, and healthcare. Sci. Rep..

[5] Koo J.H., Kim D.C., Shim H.J., Kim T.-H., Kim D.-H. (2018). Flexible and stretchable smart display: materials, fabrication, device design, and system integration. Adv. Funct. Mater..

[6] Liu Y., Pharr M., Salvatore G.A. (2017). Lab-on-Skin: A review of flexible and stretchable electronics for wearable health monitoring. ACS Nano.

[7] Cataldi P., Athanassiou A., Bayer I. (2018). Graphene nanoplatelets-based advanced materials and recent progress in sustainable applications. Appl. Sci..

[8] Li R., Li M., Su Y., Song J., Ni X. (2013). An analytical mechanics model for the island-bridge structure of stretchable electronics. Soft Matter.

[9] Suikkola J., Björninen T., Mosallaei M., Kankkunen T., Iso-Ketola P., Ukkonen L., Vanhala J., Mäntysalo M. (2016). Screen-printing fabrication and characterization of stretchable electronics. Sci. Rep..

[10] Van den Brand J., de Kok M., Koetse M., Cauwe M., Verplancke R., Bossuyt F., Jablonski M., Vanfleteren J. (2015). Flexible and stretchable electronics for wearable health devices. Solid State Electron..

[11] Zamarayeva A.M., Ostfeld A.E., Wang M., Duey J.K., Deckman I., Lechêne B.P., Davies G., Steingart D.A., Arias A.C. (2017). Flexible and stretchable power sources for wearable electronics. Sci. Adv..

[12] Mosallaei M., Jokinen J., Honkanen M., Iso-Ketola P., Vippola M., Vanhala J., Kanerva M., Mantysalo M. (2018). Geometry analysis in screen-printed stretchable interconnects. IEEE Trans. Compon. Packag. Manuf. Technol..

[13] Mosallaei M., Khorramdel B., Honkanen M., Iso-Ketola P., Vanhala J., Mantysalo M. Fabrication and characterization of screen printed stretchable carbon interconnects. Proceedings of the 2017 IMAPS Nordic Conference on Microelectronics Packaging (NordPac).

[14] Wang Y., Li Z., Xiao J. (2016). Stretchable thin film materials: fabrication, application, and mechanics. J. Electron. Packag..

[15] Cataldi P., Dussoni S., Ceseracciu L., Maggiali M., Natale L., Metta G., Athanassiou A., Bayer I.S. (2018). Carbon nanofiber versus graphene-based stretchable capacitive touch sensors for artificial electronic skin. Adv. Sci..

[16] Harris K.D., Elias A.L., Chung H.-J. (2016). Flexible electronics under strain: A review of mechanical characterization and durability enhancement strategies. J. Mater. Sci..

[17] Zhao Y., Huang X. (2017). Mechanisms and materials of flexible and stretchable skin sensors. Micromachines.

[18] Vuorinen T., Niittynen J., Kankkunen T., Kraft T.M., Mäntysalo M. (2016). Inkjet-printed graphene/PEDOT:PSS temperature sensors on a skin-conformable polyurethane substrate. Sci. Rep..

[19] Park M., Park J., Jeong U. (2014). Design of conductive composite elastomers for stretchable electronics. Nano Today.

[20] Zhang Y., Fu H., Su Y., Xu S., Cheng H., Fan J.A., Hwang K.C., Rogers J.A., Huang Y. (2013). Mechanics of ultra-stretchable self-similar serpentine interconnects. Acta Mater..

[21] Cheng T., Zhang Y., Lai W.-Y., Huang W. (2015). Stretchable thin-film electrodes for flexible electronics with high deformability and stretchability. Adv. Mater..

[22] Gonzalez M., Axisa F., Bulcke M.V., Brosteaux D., Vandevelde B., Vanfleteren J. (2008). Design of metal interconnects for stretchable electronic circuits. Microelectron. Reliab..

[23] Someya T. (2012). Stretchable Electronics.

[24] Dang W., Vinciguerra V., Lorenzelli L., Dahiya R. (2017). Printable stretchable interconnects. Flex. Print. Electron..

[25] Khondoker M.A.H., Sameoto D. (2016). Fabrication methods and applications of microstructured gallium based liquid metal alloys. Smart Mater. Struct..

[26] Varner H., Mahaffey J., Marinis T., DiBiasio C. (2017). Encapsulation of microelectronic assemblies for use in harsh environments. Int. Symp. Microelectron..

